# Extensive variation, but not local adaptation in an Australian alpine daisy

**DOI:** 10.1002/ece3.2294

**Published:** 2016-07-10

**Authors:** Megan J. Hirst, Jason P. Sexton, Ary A. Hoffmann

**Affiliations:** ^1^ Bio21 InstituteSchool of BiosciencesThe University of MelbourneParkvilleVictoria3010Australia; ^2^ Royal Botanic Gardens VictoriaBirdwood AvenueMelbourneVictoria3004Australia; ^3^ School of Natural SciencesUniversity of CaliforniaMercedCalifornia95343USA

**Keywords:** Adaptation, Australian alpine, *Brachyscome*, plasticity, reciprocal field based, seed sourcing

## Abstract

Alpine plants often occupy diverse habitats within a similar elevation range, but most research on local adaptation in these plants has focused on elevation gradients. In testing for habitat‐related local adaptation, local effects on seed quality and initial plant growth should be considered in designs that encompass multiple populations and habitats. We tested for local adaptation across alpine habitats in a morphologically variable daisy species, *Brachyscome decipiens,* in the Bogong High Plains in Victoria, Australia. We collected seed from different habitats, controlled for maternal effects through initial seed size estimates, and characterized seedling survival and growth in a field transplant experiment. We found little evidence for local adaptation for survival or plant size, based on three adaptation measures: Home versus Away, Local versus Foreign, and Sympatric versus Allopatric (SA). The SA measure controlled for planting site and population (site‐of‐origin) effects. There were significant differences due to site‐of‐origin and planting site effects. An important confounding factor was the size of plants directly after transplantation of seedlings, which had a large impact on subsequent seedling survival and growth. Initial differences in plant width and height influenced subsequent survival across the growing season but in opposing directions: wide plants had higher survival, but tall plants had lower survival. In an additional controlled garden experiment at Cranbourne Royal Botanic Gardens, site‐of‐origin effects detected in the field experiments disappeared under more benign homogeneous conditions. Although *B. decipiens* from different source areas varied significantly when grown across a range of alpine habitats, these differences did not translate into a local or habitat‐related fitness advantage. This lack of local advantage may signal weak past selection, and/or weak adaptive transgeneration (plasticity) effects.

## Introduction

Transplants and controlled common garden experiments have classically been used to test for genetic differentiation and local adaptation in plant populations (Turesson 1922; Clausen et al. 1940) and are powerful tools to test performance across habitat types, and fitness variation both within and beyond present range limits (e.g., Hiesey et al. 1971; Schemske 1984; Galen et al. 1991; Stanton and Galen 1997; Verhoeven et al. 2004; Angert and Schemske, 2005; Byars et al. 2007). For local adaptation to be detected, it should be investigated within the context of a metapopulation structure where multiple environments and populations are sampled (Kawecki and Ebert 2004; Blanquart et al. 2013). Local adaptation can be defined in terms of the difference in fitness of populations on their home sites versus their fitness when transplanted to other sites (Blanquart et al. 2013), or the more stringent condition where the home site population is superior to the average fitness of other populations transplanted to the same site (Kawecki and Ebert 2004).

Several reviews have indicated that patterns of local adaptation may not necessarily be present across metapopulations (Leimu and Fischer 2008; Hereford 2009). One challenge in using transplant experiments to detect local adaptation is that plants from various habitats may differ in performance for reasons unrelated to local adaptation (Kawecki and Ebert 2004; Leimu and Fischer 2008; Hereford 2009). When undertaking transplants, seed is normally collected from the natural populations, and seed or seedlings transplanted into experimental sites (Carter and Blair 2012; Pahl et al. 2013; Pánková et al. 2014; Torang et al. 2015). This design cannot identify maternal or transgenerational effects, which might obscure the expression of local adaptation if they override genetic differences among sites or create their own patterns of variation linked to local environmental variation. An absence of local adaptation might also reflect other processes such as differences in mycorrhizal colonization patterns (Pánková et al. 2014). These issues can be minimized by growing seed in a common garden before transplantation to minimize maternal effects (Galloway and Fenster 2000). However, this procedure becomes impractical in many cases because of the long time required for plants to mature and produce seed across two generations. Moreover, under field conditions, it is realistic to include such effects in any assessment of adaptation, given that selection in the field will act on all components of phenotypic variation (Carter and Blair 2012). Nevertheless, considering seed or seedling size can somewhat account for maternal effects (Garrido et al. 2002).

Apart from the effects of seed sources, local adaptation might also be obscured by overall differences among populations due to factors such as inbreeding. For these reasons, Blanquart et al. (2013) advocate the use of linear models and designs covering multiple populations and environments where the overall impact of habitat sites and population “quality” effects on fitness measures (e.g., due to different levels of inbreeding) can be clearly identified and separated from differences in fitness due to environmental variation.

In high mountain plants, reciprocal transplants and common garden experiments have been widely used to investigate local adaptation (e.g., Byars et al. 2007; Kawai and Kudo 2011; Torang et al. 2015). In most of these studies, the main focus has been on elevation gradients (Byars et al. 2007, 2009; Gonzalo‐Turpin et al. 2009). However, alpine environments are complex and heterogeneous at similar elevations due to factors such as local topography, aspect, and soil type (Körner 2003). Evidence for local adaptation in alpine plants based on relatively stringent criteria (Kawecki and Ebert 2004) has been found even over relatively short distances (Galen et al. 1991; Byars et al. 2007), but in some cases, there seems to be a lack of local adaptation (Leimu and Fischer 2008) and population differences may largely be driven by plastic responses (e.g., Byars and Hoffmann 2009; Sedlacek et al. 2015). This may reflect adaptive plasticity (Ghalambor et al. 2007), limited genetic variation, or perhaps overly stringent conditions being used to define local adaptation.

The Australian alpine region occupies only 0.2% of the total land mass, yet hosts high species diversity and plant endemism, reflecting a heterogeneous set of environments including sphagnum bogs, tussock grassland, shrub lands, and rocky outcrops (Williams et al. 2001, 2014). Native plant species located in the Australian Alps are almost all Australian endemics, making this a highly distinctive and diverse floristic region. However, under increased warming at higher elevation habitats and a decrease in snow cover (Hennessy et al. 2003), it is expected that local habitats will be markedly altered; areas of sphagnum bogs will decrease, tree lines will rise, and there will be an upward migration of lowland species and loss of species and communities restricted to high elevations (Worboys and Good 2011). With limited space to move up elevation gradients, the potential for local adaptation within these environments needs to be established. So far, local adaptation has been tested in a few species with mixed results, although these tests have tended to use stringent criteria to define local adaptation (i.e., based on a population having the highest fitness in its own local environment). Within these restrictions, evidence for local adaptation has been found in *Poa hiemata*, an alpine grass (Byars et al. 2007), and in alpine sedges (M'Baya 2013) but not in the alpine daisy, *Craspedia lamicola*, where plastic changes appear important (Byars and Hoffmann 2009).

Daisies belonging to the genus *Brachyscome* are a highly diverse group encompassing threatened and widespread species across a range of habitats throughout Australia including the alpine region (Short 2014). Information on local adaptation is not available for this group, although some species occur in a variety of habitats. The outbreeding species *Brachyscome decipiens* occupies a range of habitats across the alpine area, including open heathlands, tussock grasslands, and rocky grasslands (McDougall and Walsh 2007). These clearly defined habitats occupied by *B. decipiens* lead to the hypothesis that this daisy is adapted to local habitat conditions, particularly given that adaptation across small scales has been demonstrated in grasses from this region (Byars et al. 2007).

Here, we test for evidence of local adaptation in *B.decipiens* from multiple sites using a field transplant experiment and a common controlled garden experiment, and we focus on the seedling establishment and early growth phase. At this early stage of development, the mortality of alpine forbs tends to be highest (Williams et al. 2014). We asked the following questions: (1) Do local conditions and differences in plant origin affect the survival and growth of the seedlings? (2) Do plants grown from seed originating from a site perform relatively better at that site? (3) Do plants from different sites but the same overall habitat show parallel responses?

## Materials and Methods

### Study species and experiments

*Brachyscome decipiens* is one of approximately eighty species within the Australasian genus *Brachyscome*. It is a perennial scapose herb, with obovate‐oblanceolate to narrowly elliptic leaves with one to six flowering stems per rosette. It can be found in several habitat types ranging from alpine heaths and herb fields, subalpine and montane woodlands, grasslands and swamps. The flowering time for this species is dependent on locality, with earlier flowering typically occurring in the lowland areas in spring, and throughout spring into early autumn in alpine regions. *Brachyscome decipiens* is a widespread outcrossing species, with documented occurrences of polyploidy, and it is distributed across the lower eastern states of Australia from New South Wales, Australia Capital Territory, Victoria, and Tasmania. McDougall and Walsh (2007) surveyed vascular plants present within communities of the Australian Alps and found that *B. decipiens* is present in 48% of treeless alpine communities.

**Figure 1:**
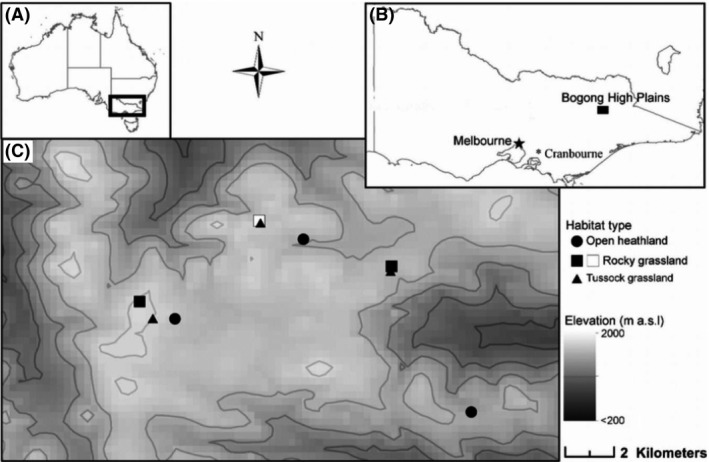
(A) Map of Australia with black outline indicating the southern state of Victoria. (B) State of Victoria, depicting the Bogong High Plains (BHP) (where the field‐based experiments were conducted) and Cranbourne Botanic Garden (where the controlled common garden experiment was conducted) in relation to the major city of Melbourne. (C) Map of the BHP indicating where the reciprocal transplants were situated. The three black symbols represent all of the eight reciprocal sites of which three are open heath land sites, two are rocky grassland, and three are tussock grassland sites. The white symbol represents a rocky grassland origin site only not an experimental planting site. Contours are 200 m.

We conducted a reciprocal field‐based transplant experiment located on the Bogong High Plains (BHP) of Victoria, and a controlled garden experiment at the Royal Botanic Gardens, Cranbourne, near Melbourne (see Fig. 1). The basic design in these experiments is similar in that seed was sourced from field sites, cleaned and cut tested to ensure high seed viability, germinated in a glasshouse, and then grown to the seedling stage before being transplanted. Seedlings were measured within 3 weeks so that any initial effects on establishment could be separated from subsequent selection.

Topography varies considerably over the Victorian BHP (as in the Australian Alps generally), with steep slopes, and broad flat valleys incurring differences on weather conditions at the local scale (McDougall and Walsh 2007). Three vegetation communities were selected based on the presence of *B*. *decipiens* and differences in topography, soil moisture, and community assemblage. Each community type had two or three replicate sites within the broader BHP area (Table S1). We followed habitat definitions from McDougall and Walsh (2007). The first habitat is the open heathland, BHP *Grevillea australis*–*Phebalium squamulosum* Community 26 (McDougall and Walsh 2007). This is the most common plant community of the BHP with high plant diversity, especially in herbs, and is primarily dominated by *G. australis* and/or *P. squamulosum* shrubs. The elevation range of this community is from 1560 to 1960 m (1775 m mean elevation range). The second habitat type is the rocky grassland *Austrodanthonia alpicola*‐*G. australis*, Community 51 (McDougall and Walsh 2007). This community occurs on cliffs and rocky outcrops with a significant proportion of shrubs and relatively low plant cover. The elevation ranges from 1420 to 2180 m (1790 m mean elevation). The third habitat type is the tussock grassland, BHP *Poa costiniana* Community 24 (McDougall and Walsh 2007). This community is dominated by dense *P. costiniana* tussocks and has lower species diversity than other alpine grasslands at similar elevations. This grassland has small depressions (which are periodically filled with water) in areas underlain by basalt (McDougall and Walsh 2007). Invasive alien plants are generally more abundant in this grassland than in others on the BHP (McDougall and Walsh 2007). The altitudinal range of this grassland is from 1570 to 1840 m (1730 m mean elevation).

The morphology of *B. decipiens* differs across these three habitat types. A survey undertaken in January 2016, measuring plant width and longest leaf across 25 localities on the BHP, confirmed these morphological differences. Measurements of 199 plants, from across eight (tussock grassland and open heathland) or nine (rocky grassland) sites for each of the three habitat types, showed that plants growing in a tussock grassland habitat were wider ($\overset{¯}{x}$ = 114 ± 42, *N* = 63) than plants growing in an open heathland ($\overset{¯}{x}$ = 95.9 ± 26.8, *N* = 64) or rocky grassland habitat ($\overset{¯}{x}$= 95.7 ± 34.9, *N* = 72). Plants growing in a tussock grassland habitat also had significantly (*P* = 0.003) longer leaves ($\overset{¯}{x}$ = 68 ± 25.7, *N* = 63) than the other two habitat types.

### Field transplant experiment (Bogong High Plains)

In order to test seedling survival and growth under natural field conditions, we collected seed of *B. decipiens* in early 2011, from within replicate sites for each habitat type. At each location, seed was randomly collected from 30 to 80 plants (20–50 seeds per plant) within a 100 m radius of each experimental site (at eight sites in total). There was one additional seed collection (and therefore site of origin) made at a rocky grassland habitat that was not used as an experimental site (due to the presence of feral horses) (Table S1). A combined seed mix from each collection was created to represent each population at that site. These collection sites represent the site of origin and the habitat of origin.

Three to five seeds from each site mix were sown into individual Jiffy^™^30‐mm pellet packs (ca. 30–50 pellets per site) (Jiffy Products International BV [Eurasia and Pacific], Moerdijk, the Netherlands) in a nursery. Germination and early cotyledon growth occurred over a 6‐week period and seedlings were thinned to allow only one seedling per plug. A solution of the seaweed‐based plant fertilizer Seasol^™^ (Bayswater, Vic., Australia) was applied to the seedlings 2 weeks postgermination. At the four‐leaf stage when seedlings were approximately 4 mm, seedlings were placed outdoors for a further 14 days to harden prior to field planting, which occurred in autumn to mirror natural germination and early development in the field (M. J. Hirst, pers. obs.).

The field experiment involved a total of 1391 seedlings planted in a completely randomized design at each locality, in each of two experimental plots per locality, with 10 × 8 cm spacing. Seedlings were watered once in the plots. We undertook the following measurements with digital calipers: plant height (based on rosette leaves), plant width, longest leaf, and plant mortality. At the conclusion of the study, we calculated biomass of the survivors by harvesting plants into paper bags kept in a cool box. Soil was removed by washing after samples were returned to the laboratory, and the aboveground shoots and root sections were weighed and bagged separately. All material was then dried in an oven at 60°C for 72 h before plants were reweighed.

In order to assess the extent to which the size of surviving plants in experimental plots differed from resident *in situ* individuals, in January 2015, we compared morphological traits and aboveground biomass of plants naturally occurring in close proximity to the experimental sites and those that were grown in the transplant experiment. We sampled 14 sites identified as *B. decipiens* localities (see Table S1). At each locality, 30 adult individuals of *B. decipiens* were selected and aboveground parts were removed and weighed twice (fresh and dried weight). Total plant width and two leaf measurements per individual were also recorded. In January 2016, we surveyed again across 25 localities measuring total plant width and longest leaf within a radius of 30 km of the field‐based experiments to ensure we captured any variation across the three habitat types: open heathland, rocky grassland, and tussock grassland.

### Controlled common garden experiment (Cranbourne)

In order to compare seedling survival and growth under homogeneous conditions, we established a common garden experiment within controlled conditions in raised outdoor planter boxes. Seeds were collected and grown to the seedling stage as for the field experiment. A total of 710 seedlings were planted across four planter boxes (plots) in a randomized block design with numbers of plants varying in each plot from 183 to 174. The soil in each box was a mix of one‐part brown coal, one‐part double‐composted mulch, and eight parts sand. Irrigation in the boxes involved 0.185 L of water per plant for two 3‐min periods separated by 10 min applied daily unless it rained. Measurements of plant height, width, and longest leaf were taken at 6‐week intervals with a digital caliper.

### Analysis

To investigate overall plant growth in the field plots, we plotted the means and ranges of plant height and width from the survey data and compared these against the height and width of plants from the transplant experiment growing at the same sites. We also looked at the growth rate of plants that survived to the end of summer by plotting changes in width and height for each of the planted sites.

To examine local adaptation for survival, we considered plants at three time periods – following transplantation, after winter, and after summer – and scored performance in terms of survival and plant size (measured as width, height, and weight at the end of the experiment). Following Blanquart et al. (2013) and using their terminology, we considered three measures of local adaptation: Home versus Away (HA), defined as mean fitness of the local population at its home site minus mean fitness at away sites; Local versus Foreign (LF), defined as mean fitness of the local population at its home site minus average fitness of away populations at the same site; and Sympatric versus Allopatric (SA), the difference in mean fitness between sympatric combinations of populations and sites (i.e., the mean of plants at their home sites) and mean fitness in allopatric combinations (i.e., the mean of plants at their away sites). HA is considered confounded by overall differences in habitat quality, whereas LF is confounded by population quality that might include factors such as inbreeding and transgenerational effects that may not be specific to a particular environment. Note that SA is related to HA, but importantly as emphasized by Blanquart et al. (2013), SA is not confounded by overall differences between sites and overall differences in population quality, which are adjusted in linear models.

We computed HA and LF for each measure of fitness and population and then compared these to 0 with *t*‐tests. This estimate was undertaken for comparisons of home sites (one local population per planting site) treating each plot within a locality separately, and also for comparisons involving habitat (multiple home and away habitats per home site). For SA, we tested whether the means of the home and away comparisons differed from each other, with site and population quality effects included in the linear models and tested directly statistically. In this test, we treated survival as a binomial variable and used generalized linear models (GLMs) run in IBM Statistical Package for the Social Sciences 22 (SPSS; IBM Corp, Armonk, NY).

We were interested in testing whether the traits measured at one time point might influence the performance of plants at a later time point, and the extent to which such effects might contribute to site‐of‐origin and planting site effects. In particular, we wanted to establish whether transplant differences in plant size between sites and associated with different origins might influence subsequent fitness. For the survival analysis, we treated survival as a binomial variable and then ran GLMs to investigate the impact of site of origin, site of planting, and their interaction on survival of the individual plants. To test whether plant size influenced survival, we assessed survival in spring and included plant size (width, height) during the transplantation census period as covariates. We repeated this analysis for survival across the summer period, considering only those plants that survived in spring. In all cases, we selected final models by excluding higher order interaction terms if these were not significant at the 0.15 level (Sokal and Rohlf 2012).

To visualize the association between plant traits taken at a particular census point on subsequent survival at the ensuing census point, we plotted trait means for groups of surviving plants from a particular origin/site against trait means of the same group that did not survive. If trait values were not associated with subsequent survival, we expected plotted points to fall on a line with unit slope. Plots were produced for spring survival (using size measures after transplantation) and summer survival (using size measures after transplantation and in spring).

## Results

### Field transplant experiment

#### Overall survival and growth

Survival decreased gradually after initial plantings and although plant morphology varied by site, plants did not achieve natural plant size during the experimental period. After being transplanted, 1105 of the 1391 plants survived (79.4%), while by spring, there were 474 seedlings left (42.9% of plants that survived transplantation). In the final census, 200 plants survived (42.2% of those alive in spring, 14.4% of starting population). Plants surviving to the end of summer had grown substantially at some sites but not at others (Figure S1A,B). However, experimental plants remained smaller than those from field sites for all traits at all sites, reflecting possible age differences, with the exception of fresh weight at one of the sites, and width/leaf length in wet tussock grassland habitats (Figure S2). The survey data also indicate differences in plant morphology among sites, but these were not necessarily consistent with habitat type (Figure S2).

#### Tests of local adaptation: Home versus Away, Local versus Foreign, Sympatric versus Allopatric

**Figure 2:**
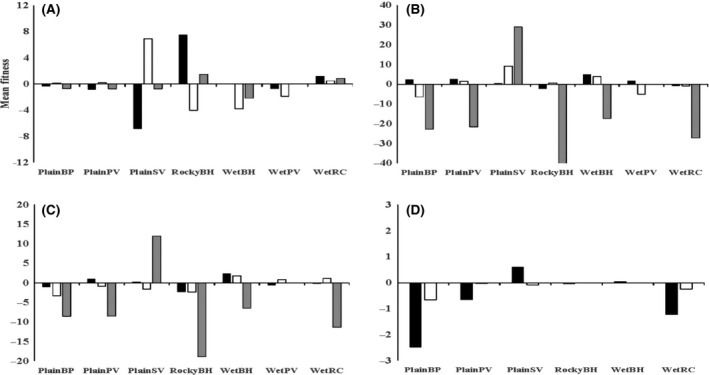
Local adaptation as measured by HA (Home vs. Away) for various fitness measures; (A) survival, (B) plant width, (C) plant height, and (D) biomass within each of the seven field sites. Positive values indicate local adaptation, whereas negative values indicate the opposite. For Figures (A), (B), and (C), transplant phase is indicated in black, spring is indicated in white, and autumn is indicated in grey. For Figure (D) fresh weight is indicated in black and dry weight is indicated in white.

**Figure 3:**
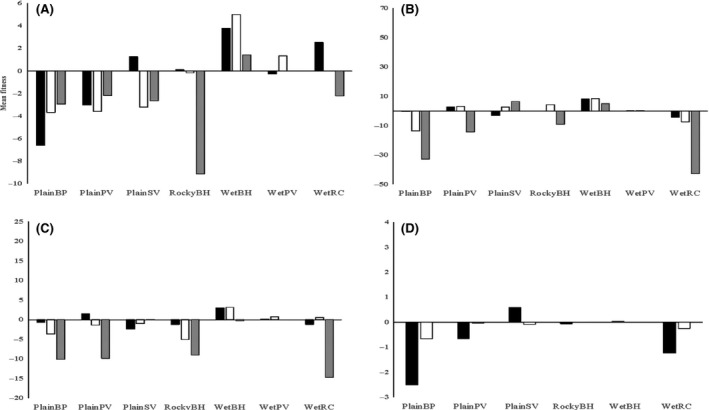
Local adaptation as measured by LF (Local vs. Foreign) for various fitness measures: (A) survival, (B) plant width, (C) plant height, and (D) biomass within each of the seven field sites. Positive values indicate local adaptation, whereas negative values indicate the opposite. For Figures (A), (B), and (C), transplant phase is indicated in black, spring is indicated in white, and autumn is indicated in grey. For Figure (D), fresh weight is indicated in black and dry weight is indicated in white.

The local adaptation analyses provided evidence of overall planting site effects and site‐of‐origin effects but no evidence of local adaptation at any of the three time points where plants were scored. We consider nine tests of local adaptation, based on three time points (at the transplant stage, over winter, and over the ensuing summer period), and three traits (survival, plant width, plant height), as well as two additional tests based on dry and wet weight at the end of the experiment. For HA (mean fitness of the local population at its home site minus mean fitness when at away sites), *t*‐tests were significant in four cases, involving width, height, and weight at the end of summer (HA ≠ 0, Table 1). However, in each case, the *t* values were negative because the local populations performed relatively worse at the local sites than at the other sites (Fig. 2). For LF (mean fitness of the local population at its home site versus average fitness of away populations at the same site), there were two significant effects, involving autumn survival and spring plant height (Table 1). However, again *t* values were negative, reflecting a lower fitness of plants at their home site compared to plants from the away sites (FL ≠ 0, Fig. 3).

**Table 1:** Test of local adaptation based on the HA (Home vs. Away) and LF (Local vs. Foreign) measures. *T*‐tests consider differences from 0 for HA and LF in the field transplant experiment scored on three occasions (transplant, spring, summer). Note that fresh weight and dry weight were only scored at the end of the experiment

Trait	Time scored	HA			LF		
		df	*t*	*P*	df	*t*	*P*
Survival	Transplant	13	0.036	0.972	13	−0.991	0.340
	Spring	13	0.276	0.787	13	−0.691	0.502
	Autumn	12	0.101	0.921	11	−2.944	0.013
Width	Transplant	13	1.311	0.212	13	0.412	0.687
	Spring	13	0.203	0.842	13	−0.142	0.889
	Autumn	13	−2.609	0.022	11	−2.086	0.061
Height	Transplant	13	−0.217	0.832	13	−0.194	0.849
	Spring	13	−0.725	0.481	13	−1.040	0.317
	Autumn	13	−2.971	0.011	11	−3.177	0.009
Fresh weight	Autumn	13	−2.329	0.037	11	−1.974	0.074
Dry weight	Autumn	12	−4.679	0.001	11	−1.778	0.103

For the SA (the difference in mean fitness between sympatric combinations of populations and sites and mean fitness in allopatric combinations) analysis (Table 2), there were significant effects of origin for six of the comparisons, and significant effects of planting site for eight comparisons, reflecting overall differences in origin and in the sites where *B. decipiens* were planted. Once these effects were controlled, we failed to find any significant effects in the HA comparison, suggesting that local adaptation was absent in this study and any differences in HA and LF were due to overall effects of planting site or site of origin.

**Table 2:** Test of local adaptation after controlling for site‐of‐origin and planting site effect. Generalized linear models testing for Sympatric versus Allopatric (SA), the difference between the average fitness in sympatric combinations and the average fitness in allopatric combinations. Differences in SA are indicated by the “Home versus Away (HA)” term, whereas the other terms test for site‐of‐origin and planting site effects. Data were considered as a binomial (survival) or continuous (other traits). For survival, significance is based on the likelihood ratio (*G*), while for the continuous variables, it is based on an *F* test

Trait	Time scored	Population (df = 7)		Planting site (df = 6/7)^a^		HA (df = 1)		Error df
		*G*/*F* b	*P*	*G*/*F*	*P*	*G*/*F*	*P*	
Survival	Transplant	75.098	<0.001	35.478	<0.001	0.097	0.756	112
	Spring	8.868	0.262	32.69	<0.001	0.043	0.836	109
	Autumn	18.522	0.010	97.18	<0.001	0.222	0.638	96
Width	Transplant	10.087	<0.001	0.891	0.517	1.432	0.234	102
	Spring	5.269	<0.001	10.943	<0.001	1.568	0.213	96
	Autumn	2.277	0.046	7.846	<0.001	0.017	0.898	42
Height	Transplant	2.973	0.007	2.269	0.035	0.002	0.964	102
	Spring	1.006	0.432	1.545	0.161	0.121	0.729	112
	Autumn	1.81	0.111	3.794	0.004	0.201	0.657	42
Biomass								
Fresh weight	Autumn	1.523	0.163	7.191	<0.001	0.751	0.387	162
Dry weight	Autumn	1.365	0.224	1.074	0.380	0.698	0.405	159

a df = 7 except for autumn (width, height) and weight traits when df = 6.

b *G* statistic for survival, *F* test for other traits.

#### Site‐of‐origin and planting site effects

**Figure 4:**
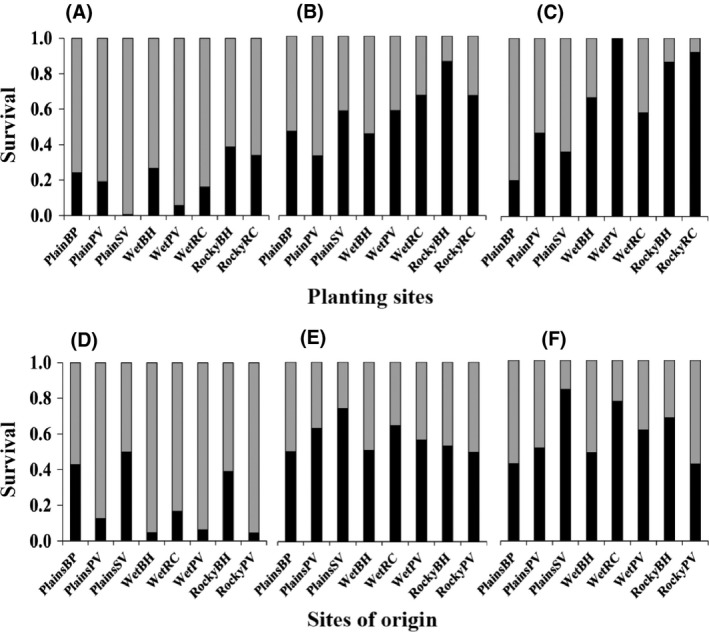
Survival of seedlings at three census points (A) after transplanting, (B) in spring and (C) in summer arranged by planting site, and (D) after transplanting, (E) in spring and (F) in summer arranged by site of origin. Stacked bars show the proportion of plants alive (grey) or dead (black). These are plotted separately for the planting sites and the sites of origin at each census point. Note that only the survivors at the transplant census point are included in the spring assessment, and only the survivors at the spring census point are included in the summer assessment.

To explore site and origin effects detected in the SA analysis in more detail, we examined the different traits across the census points. For survival, there were clear differences between sites, and also evidence that plant size scored at the transplant stage had an impact on subsequent survival. PlainSV (open heathland habitat) had the highest number of survivors after transplantation (99%), and within the same time period, RockyBH (rocky grassland habitat) showed the highest mortality (Fig. 4). The PlainPV site had the highest proportion of plants (94%) that survived between the transplant and spring census points, while the RockyBH site showed the highest mortality (Fig. 4) and planting site had previously been shown in the SA analysis to be significant in spring (Table 2). In addition, the GLM indicated that with planting site and population included in the model, both width and height of plants at the transplant stage had an impact on survival (height, *G*_1_ = 31.7, *P* < 0.001; width, *G*_1_ = 55.1, *P* < 0.001). Transplant height and width were correlated positively (r = 0.362, *P* < 0.001). However, with both variables included in the model, wide plants showed an increased probability of survival as evident from a positive slope (*b* = 0.98 ± 0.011), whereas height showed the opposite pattern (*b* = −0.106 ± 0.022); this is also apparent from comparisons of plants that survived or died across winter when means are plotted for the different planting sites and sites of origin, with width means falling below the line indicating the expected relationship if plants with a particular width had the same likelihood of dying and height means falling above the line (Figure S1; sign test, *P* < 0.01 in both cases).

In spring, differences in survival between sites became more clearly related to habitat type. Of the 441 remaining plants after the spring census, 40% survived the summer period, and planting site and origin effects showed significant effects in the SA analysis (Table 2). When transplant height and width were included in the GLM model, only width contributed significantly to survival after summer (*G*_1_ = 0.9, *P* < 0.001) and there was a positive relationship with survival (*b* = 0.117 ± 0.0272) consistent with the pattern seen in spring. PlainBP (open heathland habitat) had the highest number of survivors by the end of summer (*n* = 56), while there were no survivors at the WetPV site and mortality was high at the rocky sites (Fig. 2C). Although there was variability among the planting sites of the same habitat type, by the end of summer, survival was highest at the three open heathland (Plain) sites (Fig. 4).

**Figure 5:**
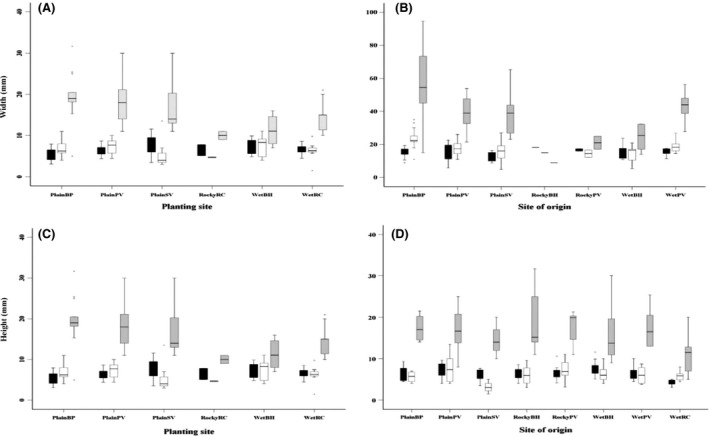
Box plots for plant width (A, B) and plant height (C, D) at three census points (after transplanting, in spring, autumn) plotted for different planting sites (A, C) and sites of origin (B, D) for the field experiment. The length of each box is the variable's interquartile range containing 50% of the data. The thick black line across each box represents the median value, the protruding lines on each box represents the smallest and largest values. Outliers (o) are values extending 1.5 box lengths from the edge of the box and extreme values (*) extend more than 3 box lengths from the edge of the box. Note due to high mortality, RockyBH and WetPV are not shown on the planting sites (A, C).

Plant width and height after transplanting and in spring showed no consistent differences linked to the habitat type of the planting sites despite site differences (Fig. 5A). After transplanting, the average width of the surviving plants was 13.04 ± 6.47 and their average height was 6.06 ± 3.32. Plants varied for both width and height with site as evident from the SA analysis (Table 2): in particular, at one of the Plain sites (PlainSV), plants tended to be relatively narrower than at the other Plain sites, and at a different Plain site (PlainBP), plants tended to be shorter than at the other sites (Fig. 5). By spring, the average width of the plants had not increased over winter at most sites with the exception of PlainBP (Figure S1), with an overall mean across all sites of 13.88 ± 10.14. The height of surviving plants had also not shifted (Figure S1) with an average of 6.07 ± 3.96. Experimental plants at one of the open heathland sites (PlainBP, $\overset{¯}{x}$ = 6.65, *N* = 48) were wider than at the other sites (Fig. 5). The site‐of‐origin effect was significant for width but not for height (Table 2).

After summer, site effects started to appear that were related to habitat type (heathland vs. tussock grassland: due to high mortality in the two rocky grassland sites, morphological measurements from these sites were not considered further). Average plant height (8.77 mm ± 5.115) increased overall and this was particularly evident at the Plain (open heathland habitat) sites (Figure S1), while average plant width (23.59 ± 14.24) also increased, again reflecting changes mostly at the Plain sites (Figures S1, 5). Planting sites were significantly different for width and height (Table 2), but origin effects had largely been lost. There was a significant difference in fresh weight across the planting sites but no other effects of weight (Table 2). Plants from the open heathland site PlainBP were the heaviest ($\overset{¯}{x}$ = 3.19 ± 2.64, *N* = 35) (Figure S1) and largest (in terms of width and height). There was no significant difference in the dry weight of plants for either the planting site, or the site of origin (Table 2).

### Controlled garden experiment

#### Survival

In this experiment, site of origin had an impact on survival but this was accounted for by differences in plant width at the transplant stage. Spring survival was high; only one seedling from 710 plants died between the transplant and the spring census phases. However, by the summer census, 65.4% of the seedlings had perished (Figure S3). Site of origin had a significant effect on survival at this time (*G*_9_ = 23.2, *P* = 0.006), and there was also a significant plot effect (*G*_3_ = 35.3, *P* < 0.001). Transplant width when included as a covariate had a significant effect on summer survival (*G*_1_ = 3.9, *P* = 0.048) and was positively associated (*b* = 0.046 ± 0.023), reflecting higher survival of wider plants. When transplant width was included in the model, site of origin was no longer significant (*G*_9_ = 8.3, *P* = 0.502), and there was also no interaction between site of origin and transplant width (*G*_9_ = 9.8, *P* = 0.363), suggesting that origin effects were mostly a consequence of differences in plant size at the start of the experiment.

#### Morphological traits

For the morphological traits, a site‐of‐origin effect was apparent initially. Only plants that remained alive at the conclusion of the experiment were used in this analysis. At the first measure taken (14 days after transplantation), the mean plant width ($\overset{¯}{x}$ = 11.5 ± 4.35, *n* = 244) was significantly affected by site of origin (*F*_9,182_ = 831.908, *P* < 0.001) (Fig. 4A), but not plot (*P* = 0.788). The widest plants were from WetBH (*N* = 37, $\overset{¯}{x}$ = 13.8 mm) and WetRC (*N* = 33, $\overset{¯}{x}$ = 13.7 mm), while plants from WetPV were narrower (*N* = 20, $\overset{¯}{x}$ = 7.8 mm). Seedlings generally had three leaves at the transplant phase, and this number was not affected by site of origin (*P* = 0.227) or experimental plot (*P* = 0.875).

**Figure 6:**
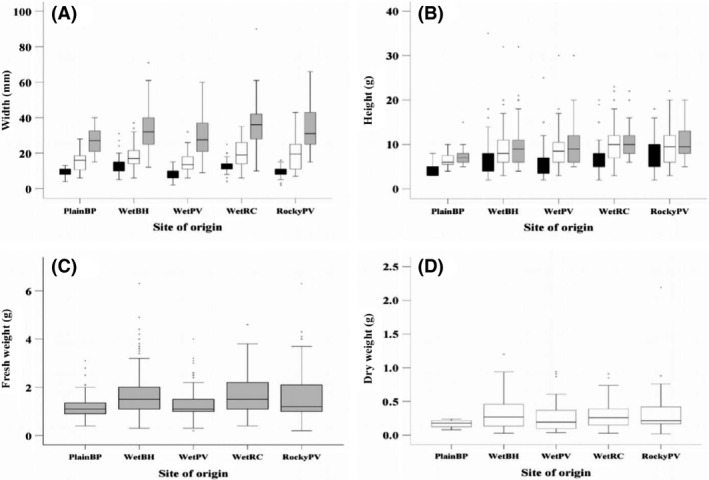
Box plots for morphological traits in the controlled common garden experiment (Cranbourne). (A) Plant width across the three census points – transplant, spring, and autumn – in a clustered variable box plot. (B) Plant height is across summer, early autumn, and late autumn. (C), fresh weight and (D) dry weight at the conclusion of the experiment plotted by site of origin.

At the later census points, a site‐of‐origin effect was no longer apparent. By spring, mean plant width had increased ($\overset{¯}{x}$ = 17.8 mm ± 7.2, *N* = 217) and plants had grown, on average, 6.2 mm since the transplant phase (Fig. 4A). There was no longer a significant effect of site‐of‐origin (*P* = 0.187) or plot (*P* = 0.318) effect. The width of the seedlings at the transplant phase included as a covariate had a significant effect on plant width in spring (*F*_1,181_ = 20.288, *P* < 0.001). By summer, mean plant width remained similar to that observed at the spring census ($\overset{¯}{x}$ = 17.7 mm ± 9.0, *N* = 217). There were no effects of site of origin (*P* = 0.532) or experimental plot (*P* = 0.629) on plant width. When transplant width was included as a covariate in the model, this covariate did not have a significant effect on plant width (*P* = 0.288), unlike in spring (Fig. 6A).

Mean plant height in summer ($\overset{¯}{x}$ = 6.7 ± 4.0, *N* = 217) was not significantly affected by the site of origin (*P* = 0.485); however, it did differ among experimental plots (*F*_3,182_ = 2.970, *P* = 0.033, plot means ranging from 4.9 to 7.5 mm). By the end of summer (March), mean plant height increased by an average of 2.8 mm (Fig. 6B, $\overset{¯}{x}$ = 9.4 ± 4.6, *N* = 217). There was no main effect of site of origin (*P* = 0.322) or experimental plot (*P* = 0.141) on height at this time. Mean plant height increased marginally from early to late autumn by 0.6 mm (May) ($\overset{¯}{x}$ = 0.97 ± 4.30, *N* = 217), and no effects of site of origin and plot were detected at this time (Fig. 6B).

There was also no evidence for site effects on plant weight measures after a year, when the final census was undertaken. Fresh weight ($\overset{¯}{x}$ = 1.25 ± 1.15, *N* = 217) was not affected by site of origin (*P* = 0.761) or experimental plot (*P* = 0.515) reflected by similar values in the box plots (Fig. 6C). Mean dry weight of the plants ($\overset{¯}{x}$ = 0.30 ± 0.26, *N* = 217) was also not affected by site of origin (Fig. 6D; *P* = 0.805) or plot (*P* = 0.754). We also examined shoot dry weight ($\overset{¯}{x}$ = 0.74 ± 0.07, *N* = 217) and found no significant effects of site of origin (*P* = 0.905) or experimental plot (*P* = 0.223) on this trait. Root dry weight ($\overset{¯}{x}$ = 0.23 ± 0.21, *N* = 217) was not influenced by site of origin (*P* = 0.720) or plot (*P* = 0.549), and neither was root length.

## Discussion

In this study, we tested for local adaptation in *B. decipiens* from different habitats, with the potential for site differences to reflect both genetic and cross‐generation effects. In our design, we were able to test many sites over similar elevation gradients, and we were able to compare field patterns to those in a common controlled garden. We found site‐of‐origin effects in the field, which may signal genetic variation or variation in transgenerational plasticity, but any variation disappeared in the *ex situ* controlled garden. No local adaptation was observed in the field. Any site differences in survival and morphology did not equate with home site advantage for populations despite large differences in traits among sites.

Our findings suggest that while plants varied among source areas that represented clearly differentiated vegetation communities (McDougall and Walsh 2007), site differences do not translate into a local fitness advantage or a habitat‐specific advantage (cf. Byars et al. 2007) regardless of which measure of local adaptation was used. Nevertheless, the effects of population and habitat on plant survival and morphology in the field are extensive and demonstrate how habitat heterogeneity translates into phenotypic variation. The absence of both home site and habitat site advantage has been noted in some previous alpine studies (e.g., Stanton and Galen 1997; Byars and Hoffmann 2009) and has been found more generally in a proportion of studies that have used field transplants to test for local adaptation (Leimu and Fischer 2008; Hereford 2009).

There are several reasons why local adaptation may not occur among plants sourced from different habitats. Local adaptation may be hindered by gene flow and/or a lack of genetic variation for traits associated with adapting to these habitats. Local adaptation has been observed in the presence of high gene flow (McGraw and Antonovics 1983), so the two are not mutually exclusive, particularly if selection pressures are strong. Nevertheless, high rates of gene flow may decrease phenotypic divergence among sites (Newman and Tallmon 2001) and reduce adaptation (Stanton and Galen 1997) particularly if gene flow acts against environmental gradients.

Although gene flow has not been directly estimated in *B. decipiens*, it is likely to be high. Seed dispersal may be unassisted and geographically limited, given that seed of *B. decipiens* lacks obvious dispersal appendages (i.e., wing‐like margins). When seed is mature, the capitulum can droop and face the soil surface rather than remain upward (Hirst, unpublished data), aiding wind dispersal (anemochory), which is a typical dispersal method for many members within Asteraceae (Andersen 1993). These features may result in patterns of gene flow that are related to distance, which is relatively common in plants (Sexton et al. 2014). Given that the different habitats we considered were patchily distributed across the BHP, we suspect that there may be a high rate of exchange of genes between the rocky, grassland, and wet areas. This is particularly likely because there is no evidence of flowering asynchrony across these habitats in *B. decipiens*. In contrast, microsatellite data from *Poa hiemata* indicate that there is more gene flow across high elevation sites from nearby peaks when compared to high and low elevation sites from the same peak (Byars et al. 2009). These patterns indicate that gene flow patterns tend to follow elevation (and environment) more closely than distance, presumably because flowering time follows elevation. This increases the likelihood of elevation‐based adaptation in this grass (Byars et al. 2007).

Low levels of local adaptation can also occur in plants that show inherently high levels of plasticity, such as in the widespread weedy European species, *Buddleja davidii* (Ebeling et al. 2011). We found morphological differences among *B. decipiens* from different populations that were linked to natural environments experienced by the plants, but these differences disappeared in the *ex situ* controlled garden experiment. This species therefore appears able to change its morphology rapidly in response to the conditions where the seedlings develop. The plastic nature of *B. decipiens* may be aided by the fact that it is a polyploid species (Solbrig et al. 1964; Smith‐White et al. 1970; Watanabe et al. 1996). Polyploidy may be a feature of species that are physiologically tolerant of fluctuating conditions and that colonize a range of environments (Comai 2005; te Beest et al. 2012).

Finally, local adaptation may not have been evident because the different conditions under which the plants were cultured failed to distinguish the performance of different genotypes. We investigated local adaptation across two growth seasons, but plants were still smaller than those in nature and these herbaceous perennials may persist at a site for several years. Local adaptation may only be evident under some conditions such as in high‐quality habitats (Hereford 2009) and may take some time to be expressed depending on longevity (Bennington et al. 2012). Nevertheless, the high level of mortality we found at our sites suggested a high potential for local selection.

The current study has also illustrated the value of carefully monitoring plants during development in field transplant experiments. Despite our efforts to start with seedlings falling into the same size class at the start of the experiment, we found that the fate of the seedlings depended critically on their early growth characteristics and that seedling width and height had opposite effects on eventual survival. This pattern suggests that any effects of seed sources on plant vigor, which are well known in a range of natural populations (Dolan 1984; Stanton 1984; Winn 1985) and agricultural contexts (Soltani and Soltani 2015), could lead to apparent source differences between sites attributable to genetic factors where none might exist.

In summary, the study has demonstrated that although plants from different source areas may vary greatly in responses when grown across a range of habitats, this does not necessarily translate into a local or habitat‐specific fitness advantage. Populations from different environments represent a collection of genotypes that may be unaffected by past environmental selection or may carry adaptive cross‐generation effects. This may signal a limited potential for adaptation, and/or high rates of gene flow, which may prevent locally adapted genotypes of *B. decipiens* developing. Conversely, *B. decipiens* may possess an “all‐purpose genotype” of which individuals enjoy a very broad niche breath (Slatyer et al. 2013). For instance, the controlled garden experiment in this study indicated that this alpine species grows well even close to sea level. In either case, we found that the natural variation observed among individuals, whether genetic or plastically derived, is important for allowing individuals to grow and thrive among different ecological conditions. In this vein, conservation of a broader set of sites and habitats increases the likelihood of producing source material that will tolerate a wider set of conditions.

## Conflict of Interest

None declared.

## Supporting information


**Figure S1.** Means plotted for the (A) width and (B) height of the different planting sites and sites of origin for data from field transplant experiment at different census points, with each group split according to whether the plants were alive or dead at the ensuing census point.
**Figure S2.** Changes in plant traits across time in the field transplant experiment.
**Figure S3.** Comparison of morphological traits in plants surviving at the end of the field transplant experiment with those measured from the surrounding vegetation at the same site.
**Figure S4**. Survival of plants by summer in the controlled common garden experiment.

**Table S1**. Additional information on the reciprocal field based experiment, the common garden and field survey; number of individuals per experiment, habitat information, and species localities with latitude (south), longitude (east) and elevation (metres).
