# Supplementary material for: Extensive variation, but not local adaptation in an Australian alpine daisy
Source: Ecol Evol. 2016 Jul 10;6(15):5459–72. doi: 10.1002/ece3.2294 (PMC4984517; doi:10.1002/ece3.2294)

# Supplementary Figures

**Supplementary Figure 1**

**Supplementary Figure 2**

**Supplementary Figure 3**


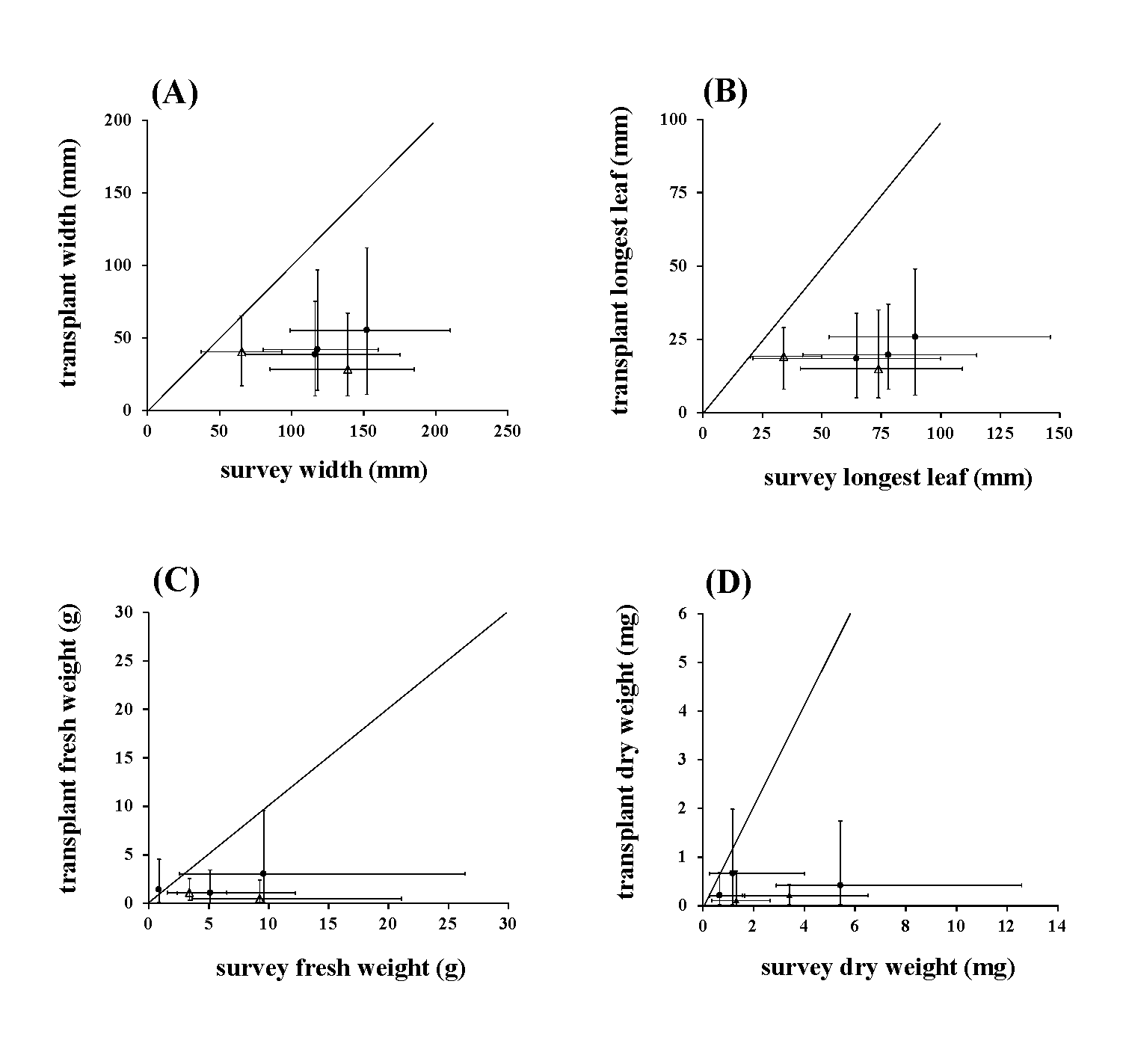


**Supplementary Figure 4**


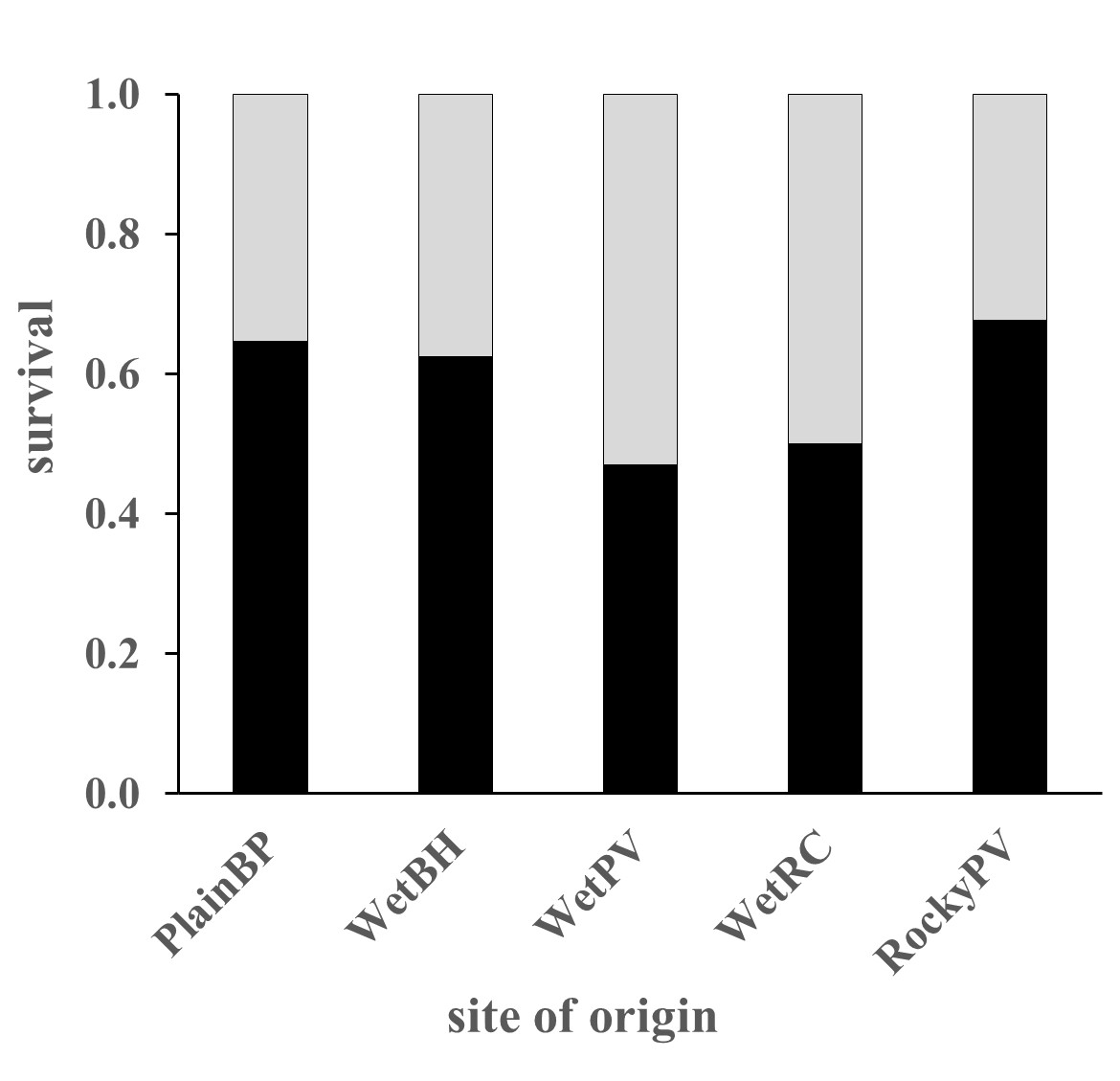

Supplement: Supplementary file 1 — Figure S1. Means plotted for the (A) width and (B) height of the different planting sites and sites of origin for data from field transplant experiment at different census points, with each group split according to whether the plants were alive or dead at the ensuing census point. Figure S2. Changes in plant traits across time in the field transplant experiment. Figure S3. Comparison of morphological traits in plants surviving at the end of the field transplant experiment with those measured from the surrounding vegetation at the same site. Figure S4. Survival of plants by summer in the controlled common garden experiment. [file ECE3-6-5459-s001.docx]
